# Annual Meetings of Hospitals

**Published:** 1921-05-14

**Authors:** 


### ANNUAL MEETINGS OF HOSPITALS.

### Queen's Hospital for Children.

The Duke of York, President of the Hospital, occupied the chair at the Annual Meeting of the Queen's Hospital for Children, Hackney Road, on May 5. Responding to a vote of thanks, his Royal Highness said that it was satisfactory to learn that the hospital was in a better financial condition than at that time last year, but renewed and energetic efforts would be needed to continue the work and to get on, possibly, with the building scheme, the need of which became more apparent every day. His Royal Highness paid a tribute to the untiring energy of the workers and to the generosity of the working men's clubs in the district. The report, presented by Lord William Cecil, Chairman of the hospital, draws attention to the necessity for giving effect to the scheme of enlargement approved by the King's Fund. The income for the year amounted to £36,302. The King's Fund made an emergency grant of £6,500, in addition to a maintenance grant of £1,500, and £4,700 was received from the National Relief Fund towards the hospital's war deficit of £7,469. The dinner at the Connaught Rooms on November 16, at which the Duke of York presided, organised to inaugurate an appeal for £25,000 for the immediate necessities of the hospital, was a great success, the total announced on that occasion being £10,000. The Children's Jewel Fund, which did such good service amongst mothers and children during the war, decided, on dissolution, to give part of the balance in hand to the hospital conditional upon a ward for ailing babies being provided with the money. The Committee accepted the gift, and have undertaken to carry out the scheme in so far as funds may be furnished for the purpose. The cost is roughly estimated at about £10,000.

### Infants' Hospital.

The report of the Infants' Hospital, submitted at the annual meeting held under the presidency of Mr. Robert Mond, the Treasurer of the institution, states that 234 new in-patients were admitted during the past year. Of those, 101 were discharged as good recoveries. 67 died, and the remainder were classified as fair, unimproved, and suffering from general diseases. The total income for the year was £6,743, and exceeded the total expenditure by £1,124. The fact that last year was the first occasion since 1913 that income balanced expenditure is attributed to emergency grants from the National Relief Fund and the King's Fund. The ordinary income is now quite inadequate, but it is hoped that some means may be found of supplementing it in order that the work of the hospital may be continued. During the year 308,233 articles were washed in the hospital laundry at a total cost of £177. Since November 1909 all the milk used in the institution has come from Coombe Bond Farm, Sevenoaks, established by the Treasurer. The diet of the cows is highly specialised, and elaborate precautions are taken to prevent contamination of the milk. Lectures on infant feeding and management are delivered at the hospital, and the income of the institution is augmented by the fees resulting therefrom.

### East London Hospital for Children.

Lord Erroll, presiding recently at the Annual Court of Governors of the East London Hospital for Children, recalled the fact that he has been connected with the institution for over thirty years. It has always been a struggle to maintain a mere sufficiency of income, which, although never equal to the expenditure, has never fallen so short as during 1920, when the income was between £12,000 and £13,000 and the expenditure £27,000. A deficit of that magnitude necessitates strong measures, but it is most regrettable to hear from the Treasurer that invested funds had to be sold to relieve the situation. Despite adverse circumstances, the Board can no longer postpone expenditure to the amount of £6,000 for preserving the building of the hospital. The funds collected by the Appeal Office were originally intended for new buildings and improvements, but, a building scheme at the present juncture being found impracticable, it has been decided, with the consent of the donors, to utilise the funds for maintenance purposes. Mr. Harry Machin, seconding the adoption of the report, drew attention to the fact that last year was started with a deficit of £1,800, whereas this year the deficit amounted to £11,000, and they have sold practically all but the Trust Funds.

